# A mixed-methods study of quality differences between applied documentation approaches in nursing homes

**DOI:** 10.1186/s12912-022-01046-2

**Published:** 2022-09-29

**Authors:** Eugenia Larjow, Madlen von Fintel, Annette Busse

**Affiliations:** 1 Department of Health Care Management, Institute of Public Health and Nursing Research, Health Sciences, University of Bremen, Grazer Str. 2a, 28359 Bremen, Germany; 2 Department of Human Sciences, Institute for Educational Science, University of Kassel, Nora-Platiel-Straße 5, 34127 Kassel, Germany

**Keywords:** Nursing documentation, Nursing home, Staff satisfaction, Quality dimensions, Structural model

## Abstract

**Background:**

Several approaches to nursing documentation exist. Some address standardised terminology and daily monitoring, whereas the structural model approach focuses on open-ended text information and special incidents. This study aims to identify quality differences between available documentation approaches from the perspectives of nursing professionals in Germany.

**Methods:**

Between October 2018 and May 2019, a convenience sample of German nursing home practitioners was surveyed concerning the quality of their documentation techniques. The quality measurement was developed from the findings of a literature review on indicators that define successful nursing documentation. Selected indicators were structured according to Donabedian’s quality dimensions of structure, process, and outcome. A mean score was calculated for each quality dimension. Non-parametric tests were employed to discover whether organisational and person-related conditions affect score values. The framework method was used to analyse textual data.

**Results:**

Responses from 250 nursing care practitioners show significant differences between users of different documentation approaches in the outcome dimension. Nurses who worked with the structural model were slightly more satisfied with their documentation approach than users of other approaches. In addition, differences between subgroups were identified depending on the mode of the tools employed for nursing documentation, participation in training, and length of time spent using the present documentation tool. Qualitative data reveal that digitalisation, unequal task distribution, and appreciation and motivation are critical topics in nursing homes.

**Conclusions:**

The results indicate that regular opportunities to reflect on challenges in documentation activities might increase nurses’ perceptions of documentation as a valuable part of nursing care. Training might serve this purpose for users of non-structural model approaches. Regardless of the specific recording techniques employed, more investment in digital infrastructure is required.

## Background

Nursing documentation is an important element of standards and principles of good nursing care provided in nursing homes. However, exactly how nursing documentation is executed is left up to each institution. In Germany, several approaches and techniques exist to meet the documentation requirements [1, 2]. Some focus on recording templates in alignment with resident’s Activities of Daily Living (ADLs) which have to be filled out each day. Other frameworks focus on documentation of special care-relevant incidents instead of documentation of repetitive nursing care activities. Some approaches are supposed to use standardised nomenclatures and checkboxes, while other approaches concentrate on narrative text-based documentation. Additionally, a selected documentation approach can be employed using electronic devices, analogue tools, or combining both electronic and paper-based materials. Regardless of the particular choice, a nursing documentation technique must ensure certain quality criteria. Differences in quality between documentation approaches can be measured, for instance, in terms of user-friendliness and staff’s satisfaction, completeness of care-relevant and person-centred information, or in terms of time required to complete nursing records [3–5].

Previous studies have discussed the advantages and challenges of employing standardised nursing terminology in nursing documentation [6–9]. Likewise, several studies have discussed how digital innovation helps to reduce documentation times but have also described barriers encountered during the implementation process [10–14]. For instance, training opportunities for nurses, time investment as well as engagement from other staff members affect the success of implementation processes of both standardised and digital documentation [15–18]. Despite this evidence, in practice, other characteristics that influence the success of a documentation approach are often in focus. In the German long-term care setting, the specific needs of nurses have been part of public discussion which supported the introduction of a new documentation approach [19]. Since 2015, nurses in Germany have been able to use a new approach to nursing documentation called the ‘Strukturmodell’ (structural model; SM). Independently from the selected mode of the tools employed for recording (e.g. electronic or paper-based), the SM aims to reform nursing documentation more generally. The new approach intends to de-bureaucratise nursing documentation by not requiring the documentation of unchanged routines. In this context, unchanged routines refer to interventions to carry out the ADLs, such as washing or dressing, as long as no relevant changes occurred compared to the latest care plan. The idea behind SM is that nursing professionals provide care even if documentation is not required. However, for some procedures, documentation remains mandatory for reasons of accounting or safety. Furthermore, the SM offers more space for nurses to use their own words to describe residents’ situations and, thus, acknowledges the expertise of nursing staff [20]. In this way, the SM seeks to reduce documentation times and improve practitioners’ attitudes towards nursing documentation. Furthermore, it aims to increase nurses’ autonomy and, as a result, their job satisfaction. Indeed, previous publications have shown that documentation times and, subsequently, the costs of recording daily routines could be reduced by opting for the SM [1, 21]. However, research is lacking whether the implementation of the new documentation approach also helps to meet further quality aspects. The present study addresses this research gap in the German nursing setting. For this purpose, the study focuses on the following research question: How do users of different documentation approaches differ in their assessments of how often their recording techniques had positive effects on their nursing practice?

## Methods

A mixed-methods research design was employed, combining primarily quantitative elements with a qualitative component. A cross-sectional survey of German nursing home staff was conducted. The questionnaire included standardised questions as well as open text fields.

Since nursing documentation fulfils multiple functions, the measurement of a documentation approach’s quality requires multidimensional assessment [22]. Therefore, we consulted Donabedian’s dimensions of structure, process, and outcome in healthcare [23]. According to Donabedian’s framework for evaluating quality in care, the assessment of health care services is not limited to outcome measures. A comprehensive evaluation of health care innovations considers also the conditions of a care situation and the process of care itself whereby the components are interdependent. In the presented study, the employed questionnaire measuring potential quality differences between documentation approaches covered all three of Donabedian’s dimensions. Furthermore, both the quantitative and qualitative analyses were based on Donabedian’s framework.

### Participants

The convenience sample comprised staff working in German nursing homes including nurse managers, trained nurses, nursing assistants, and untrained caregivers. The inclusion criteria were minimum age of 18 and experience with the documentation of nursing activities. The study recruitment involved several strategies, including invitations via posts on social media and in an online magazine and personal invitations extended during a national nursing care conference.

Nursing care providers who participated in our study were allocated into two comparison groups according to the approach they were using at the time of the survey. Group 1 consisted of users of the new SM documentation approach. Group 2 consisted of nurses who employed other (non-SM) approaches.

### Data collection

Data were collected using a self-administered anonymous questionnaire between October 2018 and June 2019. The questionnaire consisted of five subsections with relevance to the presented research question:

1. the selection of 10 subprocesses related to one of two documentation approaches, namely the SM or the non-SM;
2. questions about organisational characteristics, including the mode of the tools employed to carry out nursing documentation (paper-based, hybrid, or electronic), experience with the employed documentation approach, and options to participate in training;
3. items inquiring about quality-related aspects of documentation approaches;
4. fields for comment text; and
5. questions about personal characteristics, including age, gender, working experience, working position, and affiliation with the nursing home.

To operationalise the quality-related items of documentation approaches, we consulted two systematic reviews and referred to studies exploring the effects and outcomes of nursing documentation systems [3, 4]. We translated our findings into questions for the study questionnaire. Finally, we grouped the questions according to the quality dimensions of structure, process, and outcome [23]. For this step, we consulted the subareas for each quality dimension. The definition of the subareas was guided by Zieme [24]. Table 1 shows the allocation of the questions to the consulted subareas and the corresponding quality dimensions. For the evaluation of quality aspects, the retrieved items were queried using a five-point Likert scale. The focus of our questions was on how often the applied documentation approaches had positive effects on nursing practice. Possible responses to items were ‘never’, ‘rare’, ‘sometimes’, ‘often’, and ‘always’.

**Table 1:** Allocation of questions to the dimensions of quality in health care

Quality dimension	Subarea^a^	Question
Structure	▪ Qualification ▪ Work organisation	1. How often do you use nursing documentation to gather information about the care situation of a resident?
		2. How often does nursing documentation provide quick access to relevant information about the resident in order to prepare an up-to-date record?
		3. How often does nursing documentation help you to use standardised professional nomenclature?
		4. If you record at least partially in handwriting: How often do you find nursing documentation legible? ^b^
		5. How often do you find nursing documentation understandable?
Process	▪ Documentation in line with the care process ▪ Care organisation (holistic care, nursing rounds) ▪ Management methodology (including rota, communication)	6. How often does nursing documentation support the organisation of your care provision, e.g., as a systematic task list or as a reminder?
		7. How often does nursing documentation support you in team work, e.g., during information exchanges with colleagues and supervisors?
		8. How often does nursing documentation provide all relevant information about the nursing process of a resident?
		9. How often does nursing documentation help you to identify important care events in a timely manner?
		10. How often does nursing documentation help you to prevent a deterioration of the care situation?
Outcome	▪ Client satisfaction ▪ Employee satisfaction	11. How often does nursing documentation support you in aligning your care activities with residents’ wishes? ^b^
		12. How often do you feel that you spend too much time on nursing documentation?
		13. How often are you demotivated because of nursing documentation?
		14. Taking all these points [questions above] and your estimates of required time and costs together: How satisfied are you with the nursing documentation approach that you are using?

^a^The presented subareas were suggested by Zieme [24] who followed Donabedian’s framework for measures of the quality of care [23]

^b^Items 4 and 11 were removed from final score analysis because this improved the overall reliability with a slightly higher Cronbach’s alpha (for item 4 from 0.75 to 0.78, and for item 11 from 0.78 to 0.81). However, this reduced the total number of analysed items in the questionnaire to 12

Additionally, the questionnaire included open text fields for comments related to the positive and negative consequences of using a particular documentation approach and for recommendations on improving the current situation. The survey also asked about the time required and the costs of each employed documentation approach, which are discussed in a different publication [21]. Both user groups answered the same questionnaire.

To enhance the response rate, both paper and online versions of the survey were administered. Additionally, respondents were offered the chance to participate in a raffle to win shopping vouchers. Raffle participation was separate from the questionnaire to ensure the anonymity of the survey.

### Data analysis

Statistical Package for the Social Sciences (SPSS) version 25 was used for the quantitative analysis. Descriptive statistics (frequencies and percentages, means, and standard deviations) were calculated for participant characteristics. A mean score was computed for each of the quality subscales, that is, for the structure, process, and outcome subscales. An additional mean score for a cross-dimension scale that included all 12 items was computed to determine the comprehensive quality. The Mann-Whitney U test and the Kruskal–Wallis test were employed to examine differences in scale values between SM and non-SM users. In addition, these tests were used to determine whether scale values differed based on personal and organisational characteristics. Personal characteristics that were included in the analysis as independent variables were age, gender, years of working experience, work position, and nursing home affiliation. The independent variables that described organisational characteristics included the mode of the tools employed to carry out nursing documentation (paper-based, hybrid, or electronic), the length of experience with the employed documentation approach, and options to participate in training. Hedges *g*_s_ and Cohen’s classification of effect sizes were employed to assess the strength of detected differences between groups (small effect ≥0.2, moderate effect ≥0.5, and large effect ≥0.8; [25]). Only measures with relevant effect sizes (*g* ≥ 0.2) are reported in the Results section.

Data from open-ended questions were analysed using the framework method by Ritchie et al. [26]. The main categories of the framework were derived deductively from Donabedian’s three dimensions of healthcare quality [23]. The category ‘others’ completed the framework. Codes were developed inductively from the data and structured according to Donabedian’s quality dimensions. The initial framework consisted of 51 subcategories: 20 subcategories for the structural dimension, 17 for the process dimension, and nine for the outcome dimension.

### Validity and reliability

The quality items derived from the literature were reviewed and discussed by three academic experts. For reliability analysis, Cronbach’s alpha was calculated to assess the internal consistency of each scale. A coding guideline was developed and applied to textual data to ensure coding consistency. Two authors coded 25% of the same responses to ensure inter-coder reliability. Both also coded some of the text twice to ensure intra-coder reliability.

## Results

In total, 250 nursing practitioners provided estimates about how often their documentation techniques positively affected their nursing practices. Table 2 displays the characteristics of the sample. With 78% female participants and approximately 30% of caregivers aged between 50 and 59 years, our sample roughly corresponds to characteristics of nurses working in German nursing homes [27]. However, only 5% of participants in our sample are aged 60 and over. This age group is underrepresented compared to 13% in the German nursing staff population [27]. With 52% of respondents having a nurse manager position, a relatively large proportion of the sample represents a leadership perspective.

**Table 2:** Participant characteristics

	Structural model	Non-structural model	All documentation approaches
**Sample**	Number of responses		
Total	175	75	**250**
Scale structure	174	75	**249**
Scale process	172	73	**245**
Scale outcome	172	75	**247**
Cross-dimension scale	169	73	**242**
**Gender**	Number of responses [%] ^a^		
Female	133 [77]	58 [78]	**191 [77]**
Male	40 [23]	16 [22]	**56 [23]**
No answer	2	1	**3**
**Age**	Number of responses [%] ^a^		
Under 30 years	25 [15]	19 [26]	**44 [18]**
30–39 years	47 [28]	14 [19]	**61 [25]**
40–49 years	38 [22]	12 [16]	**50 [21]**
50–59 years	52 [30]	24 [33]	**76 [30]**
60 years and older	9 [5]	4 [6]	**13 [5]**
No answer	4	2	**6**
	Number of years		
Mean	43.5	42.1	**43.1**
*SD*	11.1	13.2	**11.8**
**Position**	Number of responses [%] ^a^		
Nurse manager	98 [57]	31 [42]	**129 [52]**
Trained nurse	71 [41]	33 [45]	**104 [42]**
Nursing assistant	3 [2]	9 [12]	**12 [5]**
Untrained caregiver	1 [1]	1 [1]	**2 [1]**
No answer	2	1	**3**
**Work experience**	Number of years since first professional qualification		
Mean	19.9	16.7	**18.9**
*SD*	11.3	12.2	**11.6**
**Nursing home affiliation**	Number of years		
Mean	10.3	8.2	**9.7**
*SD*	8.3	7.7	**8.1**
**Experience with the employed documentation approach**	Number of responses [%]		
Less than 6 months	18 [10]	3 [4]	**21 [8]**
Between 6 and 11 months	33 [19]	5 [7]	**38 [15]**
More than 11 months	124 [71]	67 [89]	**191 [76]**
**Mode of the tools employed to carry out nursing documentation**	Number of responses [%] ^a^		
Electronic	99 [57]	35 [47]	**134 [54]**
Hybrid (paper-based and electronic)	49 [28]	23 [31]	**72 [29]**
Paper-based	26 [15]	16 [22]	**42 [17]**
No answer	1	1	**2**
**Participation in training**	Number of responses [%] ^a^		
No training offered	19 [11]	27 [38]	**46 [19]**
Only one-off training	87 [51]	20 [28]	**107 [44]**
Regular training	64 [38]	24 [34]	**88 [37]**

*Abbreviation*: *SD* Standard deviation

^a^Valid percentages only

With regard to their experience in applying the surveyed documentation approaches, the overwhelming part of our study participants was quite familiar with the employed recording technique. While 71% of the SM users reported that they have been working with the new documentation approach for at least 1 year, this was true for 89% of the non-SM users. For users of the SM approach, this answer category could include a maximum period of 6 years as the SM approach was introduced in some nursing homes in 2013 in the context of a pilot project. For non-SM users, the maximum experience is indeterminate.

### Quality score

The internal consistency of the questionnaire was satisfactory for all item sets with Cronbach’s alphas between 0.78 and 0.90. Table 3 shows the Cronbach’s alphas for each scale. According to our results, SM users reported higher evaluations than non-SM users for all scales. Table 4 displays the corresponding figures.

**Table 3:** Cronbach’s alphas for item sets on quality subscales and the related comprehensive scale

Scale	Number of items	Documentation approach						
		All approaches			Structural model		Non-structural model	
		Cronbach’s alpha	Sample size	Scale mean (*SD*)	Sample size	Scale mean (*SD*)	Sample size	Scale mean (*SD*)
Structure	4	.78	*N* = 249	3.80 (0.79)	*N* = 174	3.84 (0.77)	*N* = 75	3.71 (0.83)
Process	5	.88	*N* = 245	3.45 (0.87)	*N* = 172	3.49 (0.84)	*N* = 73	3.35 (0.94)
Outcome	3	.81	*N* = 247	2.79 (0.91)	*N* = 172	2.92 (0.91)	*N* = 75	2.48 (0.85)
Cross-dimension	12	.90	*N* = 242	3.34 (0.73)	*N* = 169	3.41 (0.72)	*N* = 73	3.12 (0.73)

*Abbreviations*: *SD* Standard deviation, *N* Total number of cases

**Table 4:** Descriptive statistics and test statistics on quality subscales according to the examined documentation approach groups

	Structure		Process		Outcome		Cross-dimension	
	Structural model	Non-structural model	Structural model	Non-structural model	Structural model	Non-structural model	Structural model	Non-structural model
**Descriptive statistics**								
***Mdn***	4.00	3.75	3.60	3.20	2.67	2.33	3.42	3.01
***M***	3.84	3.71	3.49	3.35	2.92	2.48	3.41	3.18
***SD***	.77	.83	.84	.94	.91	.85	.72	.73
***N***	174	75	172	73	172	75	169	73
**Test statistics**								
***U***	5887.50		5671.00		4592.00		4963.50	
***z***	−1.22		−1.20		−3.62		−2.41	
***p***	.22		.23		<.001		<.05	

*Abbreviations*: *Mdn* Median, *M* Mean, *SD* Standard deviation, *U* Mann-Whitney U test statistic, *z* the value of a statistic divided by its standard error, *p* probability value

However, a Mann-Whitney U test only showed significant differences between the two documentation groups for the outcome scale, *U*(*N*_*SM*_ = 172, *N*_*NON-SM*_ = 75) = 4592.00, *z* = − 3.62, *p* < 0.001. The effect size was *g =* 0.49 and corresponded with a moderate effect. Likewise, significant differences between user groups were revealed for the cross-dimension scale that included all 12 items, *U*(*N*_*SM*_ = 169, *N*_*NON-SM*_ = 73) = 4963.50, *z* = − 2.41, *p* < 0.05. Here, the effect size was *g =* 0.32, which indicated a weak effect. The following sections will concentrate on these two dimensions. The reporting is focused on significant results. Higher values represent higher agreement with surveyed items.

### Open-ended answers

With regard to open-ended text entries, both groups addressed similar topics comparing the advantages and disadvantages of the documentation approaches. For example, both SM users and non-SM users reported that their documentation approaches quickly presented overviews of residents’ situations as well as events relevant to the nursing process. However, staff in both documentation groups lamented that, in their perceptions, nursing recordings only had value as documentary evidence for external authorities, for example, the medical service of the German health fund and nursing home supervisory authorities. In the following, we present three themes derived as central categories from the qualitative data: digitalisation, unequal task distribution, appreciation and motivation.

### Structure dimension

For the structural quality dimension, we identified digitalisation as a main theme in our qualitative data. Digital recording tools are an element of material equipment. The participants from both groups emphasised the advantages of these tools, such as time savings and evaluative features, but they also reported that dependence on them was a disadvantage, saying that ‘Not every person [nursing assistant] involved in care provision has prompt access to the documentation system [computer]’, and ‘IT [information technology] maintenance is very tedious and delays immediate documentation’. Furthermore, study participants cited the material lack of electronic equipment in their nursing homes leading to the ‘fight for the computer’, saying, ‘You are dependent on a device that is in a room that you have to go to first to get the information. As a rule, for a ward of 20 staff, you have just two or three PCs, which are either occupied or the idiot who was working with them before has not logged out and gone home’.

The above-mentioned conflicts of use cause losses of information: ‘since not all staff members have access to the PC or it is not used by everyone, the documentation rarely reflects all aspects of care’. Software maintenance was a further barrier for sharing up-to-date facts: ‘[There is a] restricted number of characters imposed by the software provider. Therefore, a detailed description of some care situations is not possible’.

In addition, working with electronic tools is associated with language and usage barriers. According to the data, these barriers are especially relevant for caregivers with migration backgrounds and older staff. One respondent said, ‘The older ones, many are not natives and hardly speak German, let alone write it, and many less-qualified nursing staff avoid PC work’. In addition to resulting in incomplete information, this means that ‘work is often shifted to a few’ who have computer literacy. In this context, study participants suggested explanations for electronic documentation fields in different languages, wording assistance, and text that automatically fills in via speech recognition.

Overall, our text data showed an open-mindedness in the nursing community towards the advantages of electronic documentation but also demands for investment in infrastructure. The respondents from both groups expressed that useful options would include ‘accessing the nursing data and writing directly in residents’ rooms and in public areas (via touchpad, smartphone, or something like that)’ and ‘a comprehensive internet connection and automatic speech recognition – then, information could be collected efficiently in resident’s domicile even before the admission’. Finally, some respondents mentioned that their nursing home management did not purchase available applications because of additional costs.

### Process dimension

Knowledge gaps can result in the reallocation of tasks between colleagues, part of the process dimension of Donabedian [23]. According to the reports of our study participants, documentation work was not distributed equally among staff. In the text data, this issue was linked to differing responsibilities between trained nurses and assistants: ‘Since in practice, only trained nurses are responsible for [documentation], the nursing assistants do not feel responsible for it and do not enter important information. So, some things fall by the wayside’. Another respondent said, ‘The documentation is written by professionals, but the direct contact and the main work are done by the nursing assistants’. It is important to note that a reduced amount of information to be recorded is an inherent part of the SM approach – there is no need to document daily nursing activities if they remain unchanged to the previous day’s activities. According to some statements of our participants, this characteristic of the SM motivates assistants to reduce further recording: ‘Many staff members, especially assistants, think that they no longer have to write down anything because the conventional documentation of each nursing activity is not necessary any longer. Thus, fewer reports are written by the assistants’.

### Outcome dimension

In our sample, SM users felt that they did not use too much time on care documentation. Overall, they felt to use less time on documentation than non-SM users. In addition, SM users were less demotivated due to nursing documentation and, in general, were more satisfied with their documentation approach (Table 4).

A Kruskal-Wallis test showed that the difference of the outcome scores of SM users were significantly affected by how long nurses had employed the new documentation approach, *H*(2) = 15.98, *p* < 0.001. Users who had employed the SM for more than 11 months (*Mdn* = 3.00, Table 5) had higher score values on the outcome scale than users who had worked with the new approach for a maximum of 6 months (*Mdn* = 2.33) or 11 months (*Mdn* = 2.33). Post hoc pairwise comparisons between the three groups (< 6 months; 6–11 months; > 11 months) were carried out using a Bonferroni-adjusted alpha level of 0.02 (0.05/3). Strong evidence of a difference (*p* < 0.01) was revealed between users who had used the SM for 11 months maximum and those who had used it for at least 1 year. There was a strong effect for those nurses who had used SM documentation for 6 months maximum (*g =* 0.85) and a moderate effect for those who had employed the approach for 6–11 months (*g =* 0.61). Table 5 shows the score values.

**Table 5:** Score values of structural model users according to the length of use categories

	Structural model							
	Outcome dimension				Cross-dimension			
	***Mdn***	***M***	***SD***	***N***	***Mdn***	***M***	***SD***	***N***
**Length of use in number of months**								
**< 6**	2.33	2.37	.60	17	2.88	2.97	.74	17
**6–11**	2.33	2.56	.92	33	3.26	3.27	.82	31
**> 11**^a^	3.00	3.10	.88	122	3.56	3.50	.67	121

*Abbreviations: Mdn* Median, *M* Mean, *SD* Standard deviation, *N* Total number of cases

^a^Additional information on this response category are provided in the second paragraph of the Results section

The above-mentioned tests revealed that the outcome scores were also significantly affected by the mode of the employed documentation tools, *H*(2) = 7.52, *p* < 0.05. Users of hybrid modes (paper-based and electronic, *Mdn* = 2.33) and users of electronic modes (*Mdn* = 2.67) differed significantly. This was a rather weak effect, with *g =* 0.36. Considering the specific documentation approaches, the same mode groups differed significantly for non-SM users, *H*(2) = 6.78, *p* < 0.05. The pairwise comparison showed that, after Bonferroni adjustment of the alpha level to 0.02 (0.05/3), non-SM users who recorded only with electronic tools (*Mdn* = 2.67) had a higher outcome score than nurses working with hybrid modes (*Mdn* = 2.00). This effect was moderate to strong for non-SM users (*g =* 0.72). Table 6 displays the score values of nurses working with non-SM documentation approaches according to the three modes. None of the pairwise comparisons between the recording modes were significant for SM users.

**Table 6:** Score values according to the mode of documentation tool categories

		Documentation approach							
		Non-structural model					Structural model		
		Mode of documentation tool					Mode of documentation tool		
		Paper-based	Hybrid	Electronic			Paper-based	Hybrid	Electronic
**Outcome dimension**	***Mdn***	2.00	2.00	2.67	**Cross-dimension**	***Mdn***	3.41	3.27	3.64
	***M***	2.31	2.17	2.76		***M***	3.22	3.22	3.55
	***SD***	.86	.57	.94		***SD***	.79	.71	.68
	***N***	16	23	35		**N**	25	47	96

*Abbreviations: Mdn* Median, *M* Mean, *SD* Standard deviation, *N* Total number of cases

Finally, a Kruskal–Wallis test showed a significant difference between non-SM users who took part in one-off trainings (*Mdn* = 2.00, Table 7) and those who took regular training (*Mdn* = 2.67), *H*(2) = 6.53, *p* < 0.05. With an effect size of *g =* 0.65, the independent variable of training opportunities had a moderate to strong effect on the difference of the median outcome scores between the subgroups of non-SM users.

**Table 7:** Score values of non-structural model users according to training categories with significant differences

	Documentation approach: Non-structural model		
	Training options		
	One-off training	Regular training	No training
**Outcome dimension**			
***Mdn***	2.00	2.67	2.33
***M***	2.25	2.86	2.40
***SD***	.95	.93	.65
***N***	20	24	27

*Abbreviations: Mdn* Median, *M* Mean, *SD* Standard deviation, *N* Total number of cases

Regarding the relationship between the outcome scores and personal characteristics, only gender emerged from the data as a significant attribute, *U*(*N*_Male_ = 56, *N*_Female_ = 188) = 3779.50, *z* = − 3.23, *p* < 0.001. Male nurses felt that they were spending too much time on nursing documentation less often, were less demotivated due to documentation, and were more satisfied with their documentation approaches than their female colleagues (*Mdn*_Male_ = 3.3, *Mdn*_Female_ = 2.7, higher values represent higher agreement with items from the outcome subscale). The gender effect was moderate, with *g =* 0.52. This was true for both documentation groups, SM: *U*(*N*_Male_ = 40, *N*_Female_ = 130) = 1950.50, *z* = − 2.40, *p* < 0.05, non-SM: *U*(*N*_Male_ = 16, *N*_Female_ = 58) = 299.50, *z* = − 2.18, *p* < 0.05. Table 8 shows the gender-related outcome scores according to the employed documentation approaches.

**Table 8:** Score values on outcome scale according to gender categories

	Documentation approach			
	Structural model		Non-structural model	
	Male	Female	Male	Female
**Outcome dimension**				
***Mdn***	3.33	2.67	2.67	2.33
***M***	3.22	2.82	2.94	2.35
***SD***	.94	.87	.96	.79
***N***	40	130	16	58

*Abbreviations: Mdn* Median, *M* Mean, *SD* Standard deviation, *N* Total number of cases

According to our qualitative data, whether or not nursing documentation is appreciated by peers and supervisors is linked to the outcome dimension. The SM users acknowledged that the implementation of the new approach positively affected nurses’ motivation: ‘The change to the structural model succeeded in encouraging nurses’ attention for nursing documentation’. The implementation of the new documentation approach was part of a nationwide discourse on nursing care in the professional community. Accordingly, we concluded from the text comments that if documentation is on nursing practitioners’ minds and if they are officially invited to share their experiences, this promotes positive attitudes towards documentation activities. Respondents’ comments included, ‘SIS [a subprocess of the SM] is more fun’, and ‘Basically, the structural model motivates you to work with and on documentation’. Comparable statements were missing in the non-SM group.

### Cross-dimension score

The cross-dimension scale included all 12 items of our questionnaire. Thus, it was a comprehensive quality scale, as it included structure-, process-, and outcome-related aspects of the quality construct. On the cross-dimensional quality scale, SM users perceived positive effects more often than non-SM users (*Mdn*_SM_ = 3.42, *Mdn*_NON-SM_ = 3.01, higher values represent higher agreement with the selected items; see the summary at the beginning of the Results section and Table 4).

For the cross-dimension score, a Kruskal–Wallis test provided evidence of a significant difference between user groups that had spent different lengths of time using the SM, *H*(2) = 7.97, *p* < 0.05. Using a Bonferroni-adjusted alpha level of 0.02 (0.05/3), subsequent pairwise comparisons between the three groups (< 6 months; 6–11 months; > 11 months) showed very strong evidence of differences (*p* < 0.01) between those who had used the SM for less than 6 months (*Mdn* = 2.88, Table 5) and those who had used it at least for 1 year (*Mdn* = 3.56). According to our analysis, this is a strong effect, with *g =* 0.78.

As already mentioned for the outcome score, there was a significant difference between users who employed hybrid modes (paper-based and electronic) for their recording activities (*Mdn* = 3.08) and those who only used electronic modes (*Mdn* = 3.58), *H*(2) = 15.22, *p* < 0.001. For SM users, this effect was weak to moderate (*g =* 0.48), *H*(2) = 7.71, *p* < 0.05. Table 6 presents SM users’ score values according to the employed modes of documentation tools. None of the pairwise comparisons of non-SM users revealed significant differences on the cross-dimension scale.

## Discussion

In this study, we compared the self-assessments of nurses working with different nursing documentation approaches regarding the perceived impacts of these approaches on nursing practice. One group consisted of nurses using the new SM documentation approach. The other group consisted of caregivers working with non-SM approaches. By comparing the frequency of positive effects of documentation approaches perceived by these two user groups, we identified higher scores for users of the SM approach on all three quality dimension subscales (structure, process, and outcome). This approach also had the highest scores on the cross-dimension scale including all surveyed quality items.

Furthermore, we described variables that contributed to higher staff satisfaction with the employed record-keeping practices. Concerning organisational characteristics, we found that the mode of tools employed to carry out nursing documentation and participation in training affected the outcome scores, but only for non-SM users. For SM users, the outcome scores were affected by the number of months the new approach had been used. The number of months that the SM had been used for documentation also influenced the cross-dimension scores of SM users. In addition, significant differences were found for SM users on the cross-dimension scale between groups employing different recording modes. Regarding differences in assessments based on personal characteristics, only gender was revealed as significant in the outcome scores in both documentation groups.

### Structure-related findings

Our quantitative analysis showed that nurses were more satisfied with their documentation approaches if they worked with fully digitalised tools compared to nurses who worked with hybrid modes (Table 2). Therefore, a switch to electronic documentation might support positive statements on documentation approaches. In addition, participants reported that, despite some described limitations, mobile hardware and advanced technology such as voice recognition would simplify care provision. However, a systematic review by Moore et al. [10] outlined available evidence about health information technology and found that it did not necessarily result in time savings for tasks other than documentation. In addition, Fratzke et al. [11] demonstrated that the implementation of voice recognition does not automatically lead to more satisfied staff because of new problems and decreased efficiency. However, they also illustrated that more interactive technology might contribute to more interaction between residents and caregivers because residents might ask for clarification if they hear what is being documented [11]. Thus, even if investment in voice recognition would not contribute to higher satisfaction in staff, it might favour person-centred care.

In the context of digitalisation in German nursing homes, it is important to note that investment in modern technologies by nursing home managers often fails due to a lack of financial support and incentives from the state and long-term care insurance providers. Daum and Ploch [18] pointed out that both institutions indirectly determine the budget for technological innovation. On the other hand, nursing homes are subject to a competitive healthcare market, so investment costs passed on to those in need of care could be associated with a competitive disadvantage. However, investment in satisfied staff ensures competitiveness, as it reduces turnover and professional exits.

The length of time required to use a given documentation approach is a further factor with significant impacts on nurses’ satisfaction with their recording instrument’s quality. This result is aligned with previous findings. In a study about the implementation of a computerised documentation system, Daly et al. [17] showed that it initially took longer to prepare electronic care plans but that the total required time decreased after staff learned how to work with the new system. For nursing homes with non-SM approaches that shifted to fully digitalised documentation as recommended above, this finding suggests dissatisfied staff at the beginning of the rearrangement. With regard to the steadiness of the score values of those SM users who had employed the new approach for 12 months or longer (see Table 5), our results appear to corroborate previous findings reported in the literature. Sockolow et al. [12] found a non-significant decline in clinicians’ satisfaction between 11 months and 17 months after the implementation of electronic health recording. Our results suggested a significant increase in satisfaction between 11 months maximum and 12 months or longer after the implementation of a new approach. However, given that our findings were based on a different population and a different operationalisation of satisfaction, comparisons between the two studies should be treated with caution.

Training was revealed as a significant factor for satisfaction for documentation approaches other than the SM. However, our quantitative findings did not indicate whether the impact of training should be considered in the context of literacy or as it relates to the understanding of how to document the nursing process with adequate terms. Both interpretations were the subjects of qualitative statements in the two user groups. Regarding literacy, it is important to note that about 60% of non-SM users reported not having been offered regular training opportunities. Our findings are in line with previous results by Buhtz et al. [16]. Based on a survey among German nursing trainees, the authors pointed out that, in Germany, sufficient training opportunities to gain digital expertise are still lacking in nursing education, although there is interest. Our qualitative data add to the evidence that this is an issue for postgraduate training as well. Training on how to document adequately had a positive impact on the outcome scores, coinciding with findings by Cheevakasemsook et al. [22]. These authors’ mixed-methods study in the Thai context demonstrated that nurses are educated in nursing processes but not in documentation. This lack of knowledge leads to inconsistent documentation and inadequate information available for decision-making. According to our results, both literacy and documentation training should be suitable for older employees and employees with migration backgrounds.

### Process-related findings

Our qualitative analysis revealed that informal workflow redesigns accompany the implementation of new documentation practices. This outcome was reported for both kinds of innovation – the new documentation approach and electronic recording systems. In our sample, these changes caused writing tasks to be shifted to those colleagues who were able to work with open-ended text or handle electronic tools. Previous studies examining changes after the implementation of electronic health records in clinical settings anticipate similar interdependencies: Carayon et al. [14], for instance, raised the question of whether the documentation burdens of physicians had been shifted to nurses after the implementation of electronic health recording technology in an intensive care unit. Our data add to the evidence that the introduction of innovative documentation methods in elderly care also results in the shifting of tasks between ward members. Indeed, our study revealed that nurses employ informal strategies to comply with new requirements if employees are insufficiently empowered to work with their given toolsets.

### Outcome-related findings

We found moderate evidence of gender having an influence on outcome scores. The impact of gender on nurses’ attitudes towards documentation systems is inconclusive. On the one hand, our findings generally fit with Alquraini et al. [13]. Based on a sample of nurses in Kuwaiti hospitals, the authors showed that gender is a statistically significant predictor of nurses’ attitudes towards computerised health information systems. However, in this study of a clinical setting in Kuwait, female respondents showed more positive attitudes towards a new health information system than did their male colleagues. According to our results, male nurses were more satisfied with the new documentation approach. On the other hand, gender was not significantly related to attitude in a study by Groot et al. [6]. Overall, we were surprised that gender-sensitive evidence is scarce in the area of nursing documentation. To the best of our knowledge, the recent evidence is limited to the presented study and the two cited publications. Because two of the three studies indicated that women and men significantly differed in their tendencies towards satisfaction with documentation systems, we recommend further research that examines gender-related push and pull factors for a balanced handling of documentation efforts.

Lastly, our qualitative analysis disclosed appreciation as one of the main issues that matter to nursing caregivers. This finding supports previous results by Cheevakasemsook et al. [22] and Howse and Bailey [15]. The authors highlighted that the recognition of documentation’s value by other healthcare professionals as well as by administrators is one of the factors influencing documentation quality. A further result of our qualitative appraisal was that the SM approach to documentation contributed to a higher motivation to prepare nursing records. This result corresponds with our quantitative findings: the mean value on the outcome scale, which also includes motivation, was slightly higher for SM users (*M*_SM_ = 2.92) than for users of other documentation approaches (*M*_NON-SM_ = 2.48). Additionally, our quantitative analysis showed that training might positively affect the outcome scores of non-SM users. Combining this information, regular engagement with documentation, such as during the implementation process of a new documentation framework or during regular training, positively affects staff attitudes towards nursing documentation. Therefore, it is essential for nursing homes with other approaches than the SM to offer regular opportunities for critical discussions of documentation issues. Notably, no significant differences were found for the outcome subscores if nurses just participated in one-off training. Hence, investment in regular training might help in the long term to improve motivation for documentation. This could be done, for example, during periodical meetings for nurses of all qualification levels. However, further research is necessary to develop strategies for motivating nurses at all qualification levels to participate in joint dialogue. In addition, regular communication about nursing documentation could perhaps promote feelings of appreciation for documentation.

## Limitations

This study has a number of limitations. The first is its cross-sectional design, which did not allow cause-and-effect conclusions. The second is that the selection bias of our convenience sample could have influenced the results obtained. The third is the different sample sizes of the two user groups, with a smaller number of participants in the non-SM approach group. These limitations underline the difficulty of collecting data based only on voluntary participation in the healthcare setting.

The surveyed quality aspects of nursing documentation were retrieved from systematic reviews with an older publication date. However, we selected the review by Urquhart and Currell from 2005 [4] for two reasons: the authors systematically compiled evidence from qualitative research, and they expanded the scope to practices which need free text documentation or different formats than electronic. These considerations are rarely available in the recent literature. In addition, we selected the review by Urquhart and Currell from 2009 [3] as recent literature still refers to this high-quality review which underlines the sustained key role of this work in the nursing documentation research [5].

Furthermore, the themes presented in our qualitative data were not conclusive. Further topics such as, for instance, communication were also addressed in the open-ended text responses. However, we decided to focus on the selected themes as they were also identified as subtopics in other main categories. Therefore, we anticipated that these issues deserve special attention.

Finally, both our analyses and the available literature indicated that electronic record keeping plays a dominant role in research about nursing documentation. A synthesis of statements considering only theoretical approaches to documentation without discussing the types of tools being used for writing was not possible. Thus, we cannot be sure that our findings refer exclusively to the meta-approach and do not address, at least in part, the challenges related to digitalisation.

However, despite these limitations, we believe that the outlined characteristics of users who are more satisfied with the quality of their documentation approach may help nursing home managers to critically review their documentation strategies and better evaluate the advantages and disadvantages of their current recording techniques. Furthermore, our results provide an overview of healthcare practitioners’ attitudes towards documentation in Germany.

## Conclusions

Our study shows that German nurses became more frequently aware of certain positive effects of their nursing documentation approach when they were using the structural model approach. Based on Donabedian’s framework of three quality dimensions, the difference between users was significant only on the outcome scale. The outcome scale included, for instance, nurses’ motivation. However, our findings indicate that it is not necessarily the change to a new documentation approach that improves users’ assessments of the recording instrument’s quality. Instead, investment in training and digitalisation could help to decrease barriers in documentation activities and spark better utilisation of documentation tools. This could help to balance documentation efforts between colleagues in a nursing home and improve nurses’ satisfaction with their working environments.

For better understanding of nurses’ requirements to adequate use of documentation approaches, we recommend further research. One topic for further research could be the identification of gender-sensitive needs that should be better considered in strategies to optimise nursing documentation. Another recommendation for further research concerns the development of strategies to establish appreciative and constructive communication about documentation barriers in practice.

If nursing home managers and financial authorities consider what nurses need in order to use documentation approaches adequately, this could help managers and authorities to recognise which documentation approaches best fit the customised requirements of a specific nursing home. Furthermore, this would support investment in changes that are actually necessary and contribute to positive attitudes toward nursing documentation.

## Data Availability

The datasets generated and analysed during the current study are available from the corresponding author on reasonable request.

## Abbreviations

ADLs: Activities of Daily Living
*g*: Hedges’ measure of effect size
M: Mean
Mdn: Median
non-SM: Non-Structural Model
p: Probability value
SM: Structural Model
SD: Standard Deviation
SPSS: Statistical Package for the Social Sciences
*U*: Mann-Whitney U test statistic
z: The value of a statistic divided by its standard error
